# Splitter-Based Sensors Realized via POFs Coupled by a Micro-Trench Filled with a Molecularly Imprinted Polymer

**DOI:** 10.3390/s24123928

**Published:** 2024-06-17

**Authors:** Ines Tavoletta, Francesco Arcadio, Luca Pasquale Renzullo, Giuseppe Oliva, Domenico Del Prete, Debora Verolla, Chiara Marzano, Giancarla Alberti, Maria Pesavento, Luigi Zeni, Nunzio Cennamo

**Affiliations:** 1 Department of Engineering, University of Campania Luigi Vanvitelli, Via Roma 29, 81031 Aversa, Italy; ines.tavoletta@unicampania.it (I.T.); francesco.arcadio@unicampania.it (F.A.); lucapasquale.renzullo@unicampania.it (L.P.R.); giuseppe.oliva1@studenti.unicampania.it (G.O.); domenico.delprete@unicampania.it (D.D.P.); debora.verolla@studenti.unicampania.it (D.V.); chiara.marzano@studenti.unicampania.it (C.M.); luigi.zeni@unicampania.it (L.Z.); 2 Department of Chemistry, University of Pavia, Via Taramelli 12, 27100 Pavia, Italy; giancarla.alberti@unipv.it (G.A.); maria.pesavento@unipv.it (M.P.)

**Keywords:** plastic optical fibers (POFs), molecularly imprinted polymers (MIPs), 2-furaldehyde (2-FAL), optical–chemical sensors, intensity-based sensors, POF splitter

## Abstract

An optical–chemical sensor based on two modified plastic optical fibers (POFs) and a molecularly imprinted polymer (MIP) is realized and tested for the detection of 2-furaldehyde (2-FAL). The 2-FAL measurement is a scientific topic of great interest in different application fields, such as human health and life status monitoring in power transformers. The proposed sensor is realized by using two POFs as segmented waveguides (SW) coupled through a micro-trench milled between the fibers and then filled with a specific MIP for the 2-FAL detection. The experimental results show that the developed intensity-based sensor system is highly selective and sensitive to 2-FAL detection in aqueous solutions, with a limit of detection of about 0.04 mg L^−1^. The proposed sensing approach is simple and low-cost, and it shows performance comparable to that of plasmonic MIP-based sensors present in the literature for 2-FAL detection.

## 1. Introduction

Optical biochemical sensors represent the most common type of selective sensor. In particular, optical biochemical sensors can be used to obtain ultra-sensitive and selective detection of a wide range of substances of interest, including viruses, bacteria, drugs, biomarkers, and environmental pollutants [1,2].

Fiber optic biochemical sensors are analytical devices in which a fiber optic serves as a transduction element. The first studies about glass optical fiber-based sensors date back to the second half of the 20th century [3]. Their use as probes or sensing elements is increasing in clinical, pharmaceutical, industrial, environmental monitoring, and military applications because of their excellent light delivery, long interaction length, and low cost [4,5,6,7]. The use of silica-based fibers is advantageous, as they allow single-mode waveguides, present less light attenuation, and can be used at high temperatures and in telecommunication wavelength bands. In addition, glass fibers are stable over the long term because they do not have moisture problems as polymers do [7].

In recent years, the development of new technologies made an alternative to glass possible, thanks to plastic optical fibers (POFs), which are easier to produce and to handle and can withstand smaller radii of curvature than glass [8]. Optical fiber biochemical sensors present several advantages, such as small size, versatility, remote sensing capability, and immunity to electromagnetic interference [8,9]. As a consequence, POF-based biochemical sensors have been presented to detect different substances in several application fields [10,11,12,13]. Due to their polymeric structure, POFs allow diversified configurations, such as U-bent configurations [14,15,16], tapered POFs [17], parallel polished POFs [18], and twisted tapered POFs [19]. However, POF-based sensors must be used at low temperatures (under 80 °C) and are affected by water absorption into the polymer material, and monomodal waveguides are most difficult to realize [8,9].

The most widely used optical sensors are those based on the surface plasmon resonance (SPR) phenomenon [20,21,22], but many other optical transduction techniques have been proposed [23,24], such as fluorescence [25], evanescent fields [26], interferometric techniques [27], fiber Bragg gratings (FBGs) [28,29,30,31,32], long-period fiber gratings (LPFGs) [33], and intensity-based sensors [34,35,36].

The sensing principle of intensity detection is based on the modulation of the light intensity level related to the quantity of interest. Intensity sensors can be used as structural vibration measurements to detect the breathing rate, which provides fundamental data in the diagnosis of respiratory diseases, or to monitor the cholesterol levels in the blood [34,35,36].

Usually, to detect specific substances of interest present in complex matrices, the sensing area of the optical probe is functionalized by a selective biological or chemical receptor [37]. Synthetic receptors, such as molecularly imprinted polymers (MIPs), offer numerous advantages over biological receptors in terms of stability in harsh environments and in the long term, cost-effectiveness, and the possibility of reprogramming according to the target analyte [38,39,40].

MIP-based sensors allow the detection of specific analytes by measuring the variation of physical features, such as the phase or intensity of the output signal [41,42]. These sensors are potentially useful for the detection of specific chemical markers, such as 2-furaldehyde (2-FAL), used to monitor the wear of power transformers [43].

In addition, 2-FAL is found in many types of food and beverages. In fact, this molecule belongs to the furfural family, a family of highly volatile and lipophilic organic compounds produced as intermediates of the Maillard reaction in foods when they undergo heat treatment [44]. In food matrices such as honey, beer, milk, and wine, the presence of high concentrations of furfural compounds, such as 2-FAL and 5-hydroxymethyl furfural (5-HMF), can induce deterioration of the organoleptic and nutritional properties. In particular, 2-FAL and 5-HMF production is caused by heat treatments carried out at excessively high temperatures or stress during storage [45,46,47,48,49]. Therefore, the measurement of these molecules is a criterion used to assess the freshness and quality of food and the quality of the processing and storage method used.

Furthermore, these compounds can be toxic [50]. Several studies have shown that 2-FAL and 5-HMF contained in food can react with DNA, inducing harmful mutations at the genetic level [51,52,53]. When found in food matrices in concentrations above the permitted limit, these substances can also have deleterious effects on the nervous system, the liver, and other organs of the human body [54].

For these reasons, our research group has developed several POF-based chemical sensors for the detection of 2-FAL in different matrices, such as milk [55] and wine [56].

More specifically, in [57], to improve the sensor system’s performance, a modified POF chip based on micro-holes filled with an MIP for the detection of 2-FAL is coupled with a conventional SPR chip in series, achieving an ultra-low detection limit (at the ppt level). However, in several application fields, the 2-FAL concentration of interest is at the ppb or ppm level [56].

In this work, a segmented waveguide sensor (SWS) for the detection of 2-FAL is designed and realized via POFs and MIPs. The word “segmented” comes from the fact that the optical waveguide consists of different adjacent waveguide segments (POFs) optically coupled, and at each transition between two adjacent segments, a part of the input power is transferred to the output through the guided modes, while the remaining part is transferred to the radiation modes [58,59]. Our research group used this sensing approach to realize an optical–chemical sensor for the detection of dibenzyl disulfide (DBDS) in oil [36].

Specifically, the intensity-based sensor was realized by coupling two POFs through a micro-trench made between the POFs and filled with MIPs [36]. This work presents an optical–chemical sensor for the selective detection of 2-FAL in water. For this purpose, a 2-FAL sensor is developed and tested at different analyte concentrations. Then, a selectivity test is carried out with 5-HMF to evaluate the sensor’s response in the presence of other substances different from the analyte. Finally, a comparative analysis with other 2-FAL optical sensors is presented.

## 2. Splitter-Based Sensor System

### 2.1. Splitter-Based Sensing Principle

**Figure 1:**
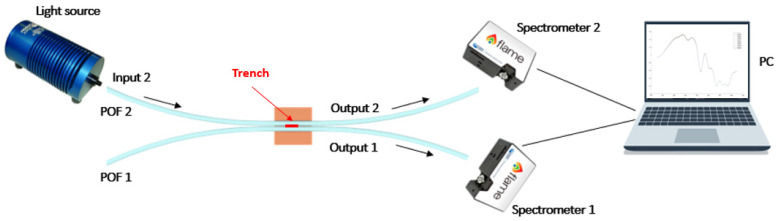
Experimental setup used to test the optical–chemical sensor based on two POFs coupled with a micro-trench filled with an MIP.

The proposed SWS consists of two adjacent waveguides (POF 1 and POF 2) optically coupled by a micro-trench that provides segmentation of the two cores, as shown in Figure 1. A white light source is connected to the POF 2, and the input power is propagated in both POF 1 and 2. In particular, the propagated power at output 2 is the direct power $\left(P_{d}\right)$ while, at output 1, the indirect power $\left(P_{i}\right)$ is propagating thanks to the coupling between the two POFs. Two spectrometers are connected to the POFs in order to collect the power contributions at different wavelengths.

The direct and indirect power rate is determined by the refractive index (RI) of the MIP present in the micro-trench, which varies with the binding between the specific MIP sites and its analyte (2-FAL) present in the tested samples (dropped over the MIP). In further detail, the MIP’s RI increases with the analyte (2-FAL) concentration in the solution. In other words, the MIP is used as a core of the waveguide between the input POF and the output POFs (POF 1 and POF 2).

In summary, it is possible to detect the concentration of the analyte by monitoring the direct and indirect output powers (at a fixed wavelength).

Consequently, the output light power at POF 1 (indirect power) rises, while the output power at POF 2 (direct power) decreases.

The direct power $P_{d}$ can be defined as [58]:

(1) $$P_{d}=F(∆n)\;P_{in}$$

where $P_{in}$ is the input power and F(∆n)  represents the transfer function of the sensor that depends on the characteristics of the micro-trench and the variation of the RI ∆n in the micro-trench.

The indirect power $P_{i}$, however, can be defined as:

(2) $$P_{i}=k\;(1-F(∆n))\;P_{in}$$

where the coefficient k depends on the number of radiation modes of the fiber POF 2 that become guided modes in POF 1.

The ratio between the two powers is:

(3) $$\frac{P_{d}}{P_{i}}=\frac{F\left(∆n\right)}{k\left(1-F\left(∆n\right)\right)}=P_{c}$$

In conclusion, the normalized analytical signal at concentration c is given as follows:

(4) $$Y_{c}=\frac{P_{c}}{P_{0}}$$

where $P_{0}$ is the ratio between direct and indirect power measured at zero concentration (without the analyte).

### 2.2. Experimental Setup

The experimental setup, shown in Figure 1, comprises several components. In further detail, the setup includes a halogen lamp (HL-2000-LL, Ocean Insight, Orlando, FL, USA), used as a white light source, coupled with one POF (POF 2) of the splitter-based sensor, along with two similar spectrometers (FLAME-S-VIS-NIR-ES, Ocean Insight) connected with the POF output of the sensor. The light source emits within the range of 360 nm to 1700 nm, while the spectrometers detect wavelengths from 350 nm to 1000 nm.

### 2.3. Splitter-Based Sensor Production Steps

**Figure 2:**
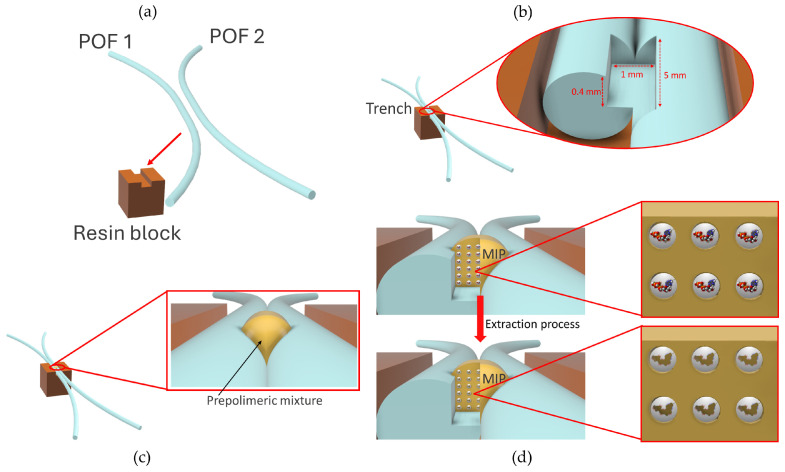
Fabrication process outline of the proposed optical–chemical sensor: (**a**) The POFs fixed in the trench of the resin block; (**b**) zoomed-in image of the trench made with a CNC machine; (**c**) focus on the MIP prepolymeric mixture dropped into the micro-trench; (**d**) MIP polymerization and template extraction processes.

As in [36], the optical–chemical chip’s fabrication process is very simple and low-cost. It consists of a few steps, summarized in Figure 2. First, two POFs, having a 980 μm PMMA core and a 10 µm fluorinated polymer cladding (1000 µm in total diameter), are fixed in parallel mode into a trench (10 mm × 2 mm × 1 mm) of a resin block in close contact (see Figure 2a). The RI values of the POF’s core and cladding are 1.49 and 1.41, respectively. The trench achieved in the resin block is 2 mm wide and 1 mm deep to accommodate two POFs. To obtain the splitter sensor, a glue (liquid cyanoacrylate, “Super Attak Loctite”) is placed in the trench before inserting two POFs. The glue adheres to the POFs’ cladding without perturbation on the propagated light.

After this, a micro-trench is made between the POFs using a computerized numerical control (CNC) micro-milling machine, as reported in Figure 2b. More specifically, a 1 mm diameter drill bit is used. A digital camera is used to align the drill bit with the POFs. The dimensions of the micro-trench (6000 µm long, 1000 µm wide, and 400 µm deep) are similar to those already published in [36]. These dimensions are a trade-off between good optical performance and mechanical integrity.

Then, the micro-trench is filled with the prepolymeric mixture to obtain the MIP sensing receptor region (see Figure 2c). In particular, a small volume (about 10 μL) of the prepolymeric mixture is dropped directly into the micro-trench, maintaining the platform in a flat position with the help of the resin support. The prepolymeric mixture expands spontaneously to fill the entire trench surface. As outlined in Figure 2d, the MIP is formed by thermal polymerization in an oven for 16 h at 80 °C [56]. Finally, the template and oligomeric polymer fragments are removed by repeated washing steps with 96% ethanol (see Figure 2d).

## 3. Materials and Methods

### 3.1. Chemicals

Divinylbenzene (DVB, CAS N. 1321-74-0), methacrylic acid (MAA, CAS N. 79-41-4), 2-furaldehyde (2-FAL, CAS N. 98-01-1), and 2,20-azobisisobutyronitrile (AIBN, CAS N. 78-67-1), were obtained from Sigma-Aldrich. All the reagents were of analytical grade, but MAA and DVB contained stabilizers in order to prevent polymerization; accordingly, they were purified with molecular sieves (Sigma-Aldrich cod. 208604, St. Louis, MO, USA) before use. Pure water was obtained using a Milli-Q system (Merck Millipore, Billerica, MA, USA). Stock solutions of 2-FAL were prepared daily by weighing the liquid and dissolving it in pure water.

### 3.2. MIP Prepolymeric Mixture

Divinylbenzene (DVB) works not only as a cross-linker but also as a solvent [56]. Thus, a proper volume of DVB was placed in a cuvette, and then the functional monomer (methacrylic acid, MAA) and the template, 2-FAL, were added to the cuvette and dissolved by sonication. The molar ratio was 1 (2-FAL):4 (MAA):40 (DVB). The mixture was uniformly dispersed and de-aerated with nitrogen for 10 min. Finally, the radical initiator AIBN (23 mg/mL of prepolymeric mixture) was added to the mixture.

### 3.3. Measurement Protocol

In order to test the proposed splitter-based 2-FAL sensor, standard solutions were prepared at different concentrations of 2-FAL: [0.01; 0.05; 0.1; 0.5; 1; 5; 10] mg L^−1^. The experimental tests were carried out by dropping 50 µL of the sample solution on the sensor’s sensing surface for 10 min (incubation time) in order to allow binding between the analyte and the MIP sites, as described in [56]. After the incubation time, the sensing area was washed three times with purified water to remove any non-specific binding. At the end of the washing steps, 50 µL of purified water was deposited upon the MIP to achieve the same bulk solution, and then the spectrum was acquired. This process was repeated for all the 2-FAL concentrations.

The power propagated to direct output was normalized to the indirect output, at different wavelengths, in order to obtain the sensor’s optical response $P_{c}$. MATLAB 2023a software was employed for data processing.

## 4. Experimental Results

### 4.1. MIP-Analyte Binding Tests

The realized optical–chemical sensor was tested with several solutions, whose 2-FAL concentrations ranged from 0.01 to 10 mg L^−1^, as described in Section 3.3. The analytical signal at concentration c ($Y_{c}$) was obtained by the normalization of the sensor response ($P_{c}$) with the blank response ($P_{0}$), at different wavelengths, as described for other intensity-based sensors [36]. In this work, with respect to [36], the experimental results achieved at 530 nm and 622 nm are reported to use wavelengths at which commercial LEDs emit.

**Figure 3:**
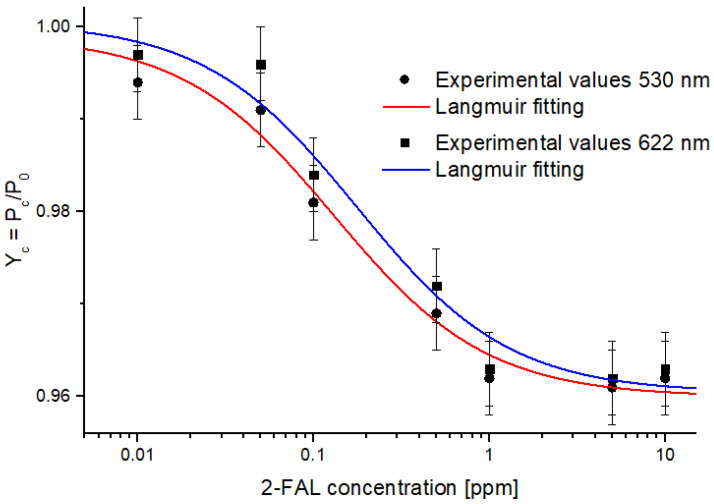
Experimental values (black markers) of the normalized signal $Y_{c}$ versus 2-FAL concentration for two different wavelengths, together with Langmuir fitting (solid lines) and error bars.

In a narrow range around these wavelengths, the $Y_{c}$ decreases when 2-FAL concentration increases. This behavior demonstrates that the sensor is sensitive to the analyte concentration variations. This phenomenon can be attributed to the RI variation of the MIP present in the micro-trench. For each wavelength of interest, dose–response curves were generated, as shown in Figure 3, where the reduction in the $Y_{c}$ intensity is a function of the increase in the 2-FAL concentration value in the sample being tested.

In particular, Figure 3 shows the experimental values of $Y_{c}$ (black markers) and relative error bars for the two considered wavelengths (530 and 622 nm).

The error bars correspond to the standard deviation obtained by repeating the measurements ten times in the same external conditions (water without analyte over the MIP).

These experimental values show a nonlinear relationship and are well fitted (solid line) using the Langmuir model, as reported in Equation (5). Specifically, the Langmuir fitting describes the receptor–analyte binding and accurately describes the sensor behavior for the variation of 2-FAL concentrations.

(5) $$Y_{c}=Y_{0}+∆Y_{max}\;\frac{c}{k+c}\;$$

In $Y_{c}\;$, c represents the analyte concentration of the solution being tested; $Y_{0}$ is the signal at 0 concentration of analyte, and based on Equation (4) ($Y_{0}=P_{0}/P_{0})$ it must be equal to 1; $∆Y_{max}$ is the maximum in terms of variation between the saturation value and the blank value of the signal; and finally, k is the dissociation constant of the aggregate target-recognition sites of the MIP. For low values of concentration (k much greater than c), Equation (5) has a linear trend, and in this case, the ratio between $∆Y_{max}$ and k can be defined as the sensitivity at low concentrations ($S_{0})$. Another parameter of interest is the limit of detection (LOD), which can be estimated as the ratio between 3.3 times the standard deviation related to the blank and the sensitivity at low concentrations. Finally, the affinity constant $K_{aff}$ can be defined as the reciprocal of the dissociation constant (*k*).

Table 1 reports the Langmuir fitting parameters obtained using OriginPro 9 software (Origin Lab. Corp., Northampton, MA, USA) relative to Figure 3.

As previously described, the binding parameters of interest can be obtained via the Langmuir fitting parameters, as summarized in Table 2, which describes, at the chosen wavelengths (530 and 622 nm), a comparative analysis of the sensor performance in terms of $S_{0}$, LOD, and $K_{aff}$.

The dose–response curves reported in Figure 3 are very similar, confirming the effectiveness of the splitter-based sensor for 2-FAL detection in aqueous solutions. Therefore, both of the chosen wavelengths are equivalent (see Table 2).

### 4.2. Selectivity Test

In order to test the selectivity of the developed optical–chemical sensor, a test was performed to observe the sensor response in the presence of an interferent, such as 5-hydroxymethyl furfural (5-HMF), by considering only one wavelength (530 nm). During the test, the signal from a 5-HMF solution at a concentration of 100 mg L^−1^ was compared with that obtained by considering a 2-FAL solution at 10 mg L^−1^.

**Figure 4:**
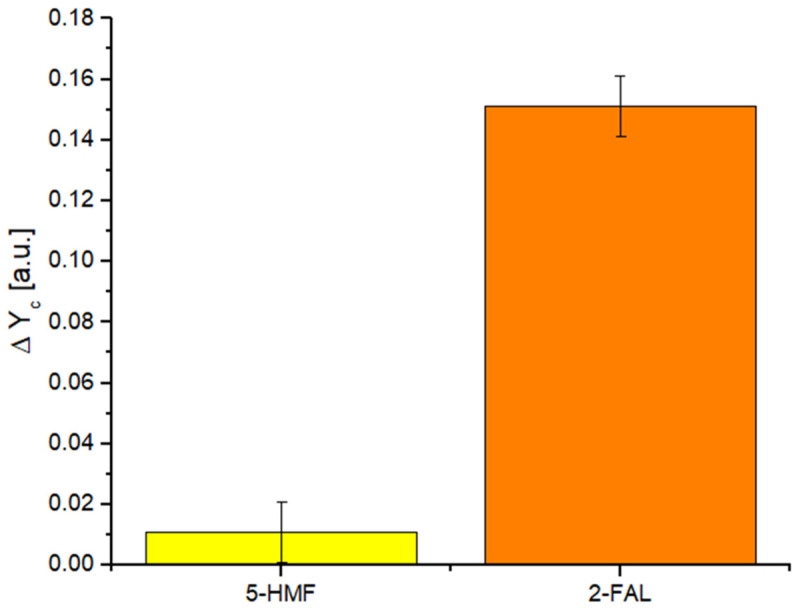
Selectivity test: $Y_{c}$ variation for 5-HMF at 100 mg L^−1^ and 2-FAL at 10 mg L^−1^.

Figure 4 shows that the 5-HMF solution (interferent) causes an insignificant variation in intensity compared to that obtained for the 2-FAL solution, even though the concentration of 5-HMF is one order of magnitude higher than that of 2-FAL.

### 4.3. Discussion

The experimental results obtained by exploiting the proposed splitter-based sensor allow a direct comparison with other plasmonic-based 2-FAL sensors already presented by our research group. Table 3 shows several sensors developed for measuring 2-FAL in different matrices, with their respective LOD values, exploiting optical transducers combined with the same MIP receptor.

As summarized in Table 3, the proposed splitter-based sensor appears to have a detection limit similar to those of most plasmonic devices already published [56,61,62,63,64].

In particular, the sensor performance is comparable to the SPR–POF platform in which the MIP is used as a receptor layer above the gold surface [56], as shown in both Table 3 and Table 4.

Specifically, Table 4 shows that the obtained similitude agrees with what was achieved for the detection of DBDS [36,65].

**Figure 5:**
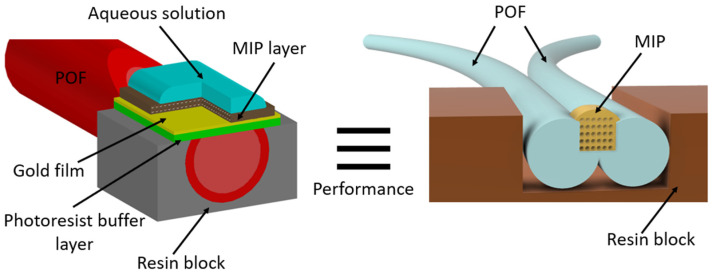
Outline of the cross-sections of the two equivalent MIP-based POF sensors. The conventional SPR–POF–MIP is depicted on the left, whereas the splitter-based sensor is depicted on the right.

More specifically, the splitter POF–MIP system is fully equivalent, in terms of LOD and K_aff_, to the conventional SPR–POF probe covered by an MIP layer (see Table 4). Figure 5 summarizes this equivalence between the proposed sensor system and the SPR–POF–MIP sensors. However, the proposed sensor performs excellently without plasmonic phenomena, reducing production costs and fabrication steps. In particular, in contrast to conventional MIP-based SPR–POF sensors [56], no polishing, spinning, or sputtering processes are required here. Similarly, in contrast to MIP-based inkjet-printed plasmonic sensors [61,62,63], no spinning or printing processes are needed.

Moreover, this kind of optical–chemical sensor, based on micromachined POFs and MIPs, overcomes existing POFs’ limitations. For instance, the solution being tested is dropped into the sensitive zone on the MIP surface; therefore, the polymeric material of the POFs does not suffer from limitations related to the phenomenon of water absorption. In the same way, the sensor’s production steps can be achieves at low temperatures, and by exploiting the MIP as a molecular recognition element, several advantages compared to bio-receptors can be achieved, including stability in the long term and out of the native environment, high selectivity, reproducibility on an industrial scale, low cost, and the regeneration possibility offered.

## 5. Conclusions

In this work, an optical–chemical sensor based on POFs and MIPs is developed and tested to detect 2-FAL, a crucial target analyte in various contexts, such as human health and power transformer life monitoring.

The presented intensity-based sensor is simple to realize, low in cost, small in size, and highly selective in 2-FAL detection. The proposed platform based on micromachined POFs can be combined with MIP receptors in order to achieve high performance without exploiting plasmonic phenomena, simplifying the fabrication process and reducing production costs. In fact, the experimental results show LOD and $K_{aff}$ values comparable to those obtained when investigating other plasmonic-based sensor configurations. Moreover, the proposed splitter-based sensor could be used as a cheap disposable chip for chemical measurements in several application fields.

In the future, the performance of these splitter-based sensors could be improved by performing an optimization analysis in terms of the geometric dimensions of the micro-trench and by employing an even more economical experimental setup based on the use of commercial LEDs as a light source and photodetectors instead of spectrometers.

## Figures and Tables

**Table 1:** Langmuir fitting parameters for 2-FAL detection in aqueous solutions.

λ [nm]	$\mathrm{START}\;Y_{0}$ [a.u]		$\mathrm{END}\;∆Y_{max}$ [a.u]		*K* [mg L^−1^]		STATISTICS	
	Value	Standard Error	Value	Standard Error	Value	Standard Error	Reduced C	Adj. R-Square
530	0.999	0.004	0.960	0.001	0.13	0.03	0.304	0.98
622	1.000	0.004	0.960	0.002	0.18	0.05	0.520	0.97

**Table 2:** Binding parameters of the optical–chemical sensor tested at the two wavelengths of interest.

λ [nm]	$S_{0}$ $\left[\frac{a.u.}{mgL^{-1}}\right]$	LOD [mg L^−1^]	$K_{aff}$ [mg^−1^ L]
530	0.297	0.040	7.60
622	0.227	0.053	5.65

**Table 3:** Comparative analysis between optical sensors based on different technologies for the 2-FAL detection.

Sensor	Matrix	$\mathrm{LOD}\;[µg\;L^{-1}]$	Ref.
MIP-filled three-micro-hole SPR–POF sensor	Milk	0.01	[55]
MIP-filled single-micro-hole SPR–POF sensor	Water	0.04	[57]
MIP-filled three-micro-hole SPR–POF sensor	Water	0.2	[57]
SPR–POF–MIP sensor	Wine	4	[56]
SPR–POF–MIP sensor	Oil	9	[60]
SPR–POF–MIP sensor	Water	43	[56]
Inkjet-printed platform (longitudinal configuration)	Water	30	[61]
Inkjet-printed platform (oblique configuration)	Water	40	[62]
Inkjet-printed platform (orthogonal configuration)	Water	50	[63]
SPR–slab-MIP sensor	Water	30	[64]
Splitter-based sensor	Water	40	[This work]

**Table 4:** Equivalence analysis between SPR–POF–MIP and splitter-based sensors for the selective detection of a specific analyte.

Sensor	Analyte	Matrix	LOD	*K*_aff_	Ref.
SPR–POF–MIP sensor	DBDS	Oil	0.01 mg L^−1^, 2.94 × 10^−8^ M	1 mg^−1^ L, 3.5 × 10^6^ M^−1^	[65]
Optical–chemical splitter sensor	DBDS	Oil	0.013 mg L^−1^, 5.3 × 10^−8^ M	2 mg^−1^ L, 8.8 × 10^6^ M^−1^	[36]
SPR–POF–MIP sensor	2-FAL	Water	0.047 mg L^−1^, 49 × 10^−8^ M	9 mg^−1^ L, 9 × 10^5^ M^−1^	[56]
Optical–chemical splitter sensor	2-FAL	Water	0.04 mg L^−1^, 48 × 10^−8^ M	8 mg^−1^ L, 8 × 10^5^ M^−1^	[This work]

## Data Availability

The data are available on reasonable request from the corresponding author.
